# Dysregulated interferon signaling and hyperinflammation upon *FCGBP* knockdown in goat bronchial epithelial cells infected with *Pasteurella multocida*

**DOI:** 10.3389/fvets.2026.1761935

**Published:** 2026-03-02

**Authors:** Yingxue Yang, Xiangyin Chen, Ziying Wang, Lili Wu, Li Du, Hongyan Gao, Si Chen, Qiaoling Chen, Fengyang Wang

**Affiliations:** Hainan Key Laboratory of Tropical Animal Reproduction & Breeding and Epidemic Disease Research, Animal Genetic Engineering Key Laboratory of Haikou, College of Tropical Agriculture and Forestry, Hainan University, Haikou, China

**Keywords:** *Fcgbp*, goat bronchial epithelial cells, Inflammation, *Pasteurella multocida*, RNA-Seq

## Abstract

**Introduction:**

The Fc gamma binding protein (FCGBP) is pivotal for mucosal immune defense against *Pasteurella multocida* (*P. multocida*), a key pathogen that induces respiratory ailments in ruminants. However, the mechanism by which FCGBP modulates the pulmonary immune response following infection remains unclear.

**Methods:**

This study investigated the role of FCGBP in the immune response of goat bronchial epithelial cells against *P. multocida* infection. An in vitro infection model was established using FCGBP-knockdown goat bronchial epithelial cells infected with *P. multocida* serotype D, and transcriptome sequencing coupled with bioinformatics was then used to detect differentially expressed genes (DEGs) and elucidate their regulatory networks. The expression levels of key DEGs and inflammatory factors were subsequently validated via quantitative real-time PCR (qRT-PCR) and enzyme-linked immunosorbent assay (ELISA).

**Results and discussion:**

The results indicated that FCGBP maintains homeostasis and provides immune protection in goat lungs. Knockdown of FCGBP in goat bronchial epithelial cells leads to impaired cellular barrier function, resulting in excessive activation of type I interferon, HIF-1, TNF, and NOD-like receptor signaling pathways following infection with *P. multocida*. These findings offer a foundational framework for elucidating the immune function of FCGBP in goat bronchial epithelial cells infected with *P. multocida*.

## 1 Introduction

FCGBP is a glycoprotein predominantly secreted by mucosal epithelial cells (1). Research has shown that FCGBP binds to the Fc region of the immunoglobulin G (IgG) antibody (2) and is regulated by cytokines and various signaling pathways, thereby mediating immune responses (1) and playing a vital role in mucosal defense (3). Specifically, FCGBP binds to trefoil factor family 3 (TFF3) to form a functional complex that contributes to innate immunity (4). During bacterial infection, the activation of Toll-like receptors (TLRs) promotes mucus secretion via the NF-κB pathway, consequently upregulating FCGBP secretion (5). Additionally, the *FCGBP* gene can enhance *IL-6* expression through the NF-κB pathway, which subsequently activates the JAK/STAT3 signaling cascade to further modulate immunity (6). It has also been suggested that FCGBP facilitates the transport of IgG to mucosal surfaces during pulmonary infection or epithelial injury, thereby preventing systemic invasion (3). However, the function of FCGBP in ruminant respiratory infections, specifically in *Pasteurella multocida* infection, remains poorly understood.

*P. multocida* is a small, coccobacillary-shaped gram-negative bacterium with an average size of 0.3–1.0 by 1.0–2.0 μm (7), and five primary capsular serotypes (A, B, D, E, and F) have been identified so far (8). This pathogen infects a wide range of hosts, including poultry, wildlife, humans, and ruminants (9). It can cause bronchopneumonia and systemic pasteurellosis in goats, resulting in substantial economic losses in animal husbandry and posing a risk to public health (10). Goats are particularly susceptible to serotypes A and D, with the latter resulting in more severe pathological damage, primarily hemorrhagic septicemia and pneumonia (11). At the molecular level, *P. multocida* infection has been shown to upregulate the expression of proteins HIF-1α and VEGFA and activate the NF-κB signaling pathway (12). Furthermore, IFN-γ plays a critical role in exacerbating toxin-associated pneumonia caused by *P. multocida*, as its deficiency has been shown to attenuate lung tissue injury (13). Research by Xu et al. (14) also demonstrated that infection activates the PI3K-Akt and FoxO signaling pathways, while another study revealed that it triggers a significant increase in key proinflammatory cytokines, such as IFN-γ, IL-12, IL-23, and IL-17 (15).

In a previous investigation, we performed transcriptome sequencing of lung tissue harvested from goats infected with *P. multocida* serotype D. Further analysis of this dataset revealed upregulation of *FCGBP* in the lungs following *P. multocida* infection (16); nonetheless, the specific role of FCGBP and underlying mechanism during infection with this P. multocida serotype remain unclear. Therefore, this study aimed to elucidate the impact of FCGBP deficiency on the immune regulatory functions of goat bronchial epithelial cells by establishing an FCGBP-knockdown model via lentiviral-mediated technology and challenging it with *P. multocida* serotype D *in vitro*. The focus was to specifically assess alterations in inflammatory cytokine expression, signaling pathway activation, and antimicrobial immune responses. The results of this study clarify the molecular basis of FCGBP-driven modulation of the cellular response to *P. multocida* infection, revealing a new molecular target for tackling relevant respiratory diseases.

## 2 Materials and methods

### 2.1 Bacterial culture

The *P. multocida* strain used in our experiments was strain HN01, serotype D (GenBank: CP037861.1), originally isolated from lung tissue obtained from a Hainan black goat and preserved in our laboratory. The bacterial culture was grown in tryptic soy broth containing 5% newborn calf serum (Sijiqing, China).

### 2.2 Cell culture and optimal multiplicity of infection for lentiviral transduction

Goat bronchial epithelial cells (Hefei Wanwu Biotechnology Co., Ltd., China) were cultured in a specialized medium containing 6% fetal bovine serum and 1% penicillin–streptomycin and maintained under standard conditions at 37 °C in a humidified 5% CO₂ incubator.

For lentiviral packaging, two *FCGBP*-shRNA lentiviral vectors (pLV3tltr-ZsGreen-Puro-U6-*FCGBP-*i1 and pLV3tltr-ZsGreen-Puro-U6-*FCGBP-*i2) and a negative control vector (pLV3tltr-ZsGreen-Puro-U6-NC) were co-transduced with two packaging plasmids into 293T cells using Lipo293™ transduction reagent (Beyotime Biotechnology, China). After 48 h of transduction, the viral supernatant was harvested and sterile-filtered through a 0.45-μm membrane and concentrated with PEG to obtain high-titer viral stocks. The viral titer was determined using the serial dilution method and calculated based on the number of cells positive for green fluorescent protein (GFP) observed under a fluorescence microscope. Calculations revealed a final titer of 5.3 × 10^8^ TU/mL for *FCGBP*-shRNA-i1, 5.5 × 10^8^ TU/mL for *FCGBP*-shRNA-i2, and 5.1 × 10^8^ TU/mL for NC-shRNA. To determine the optimal multiplicity of infection (MOI), goat bronchial epithelial cells were distributed into 96-well plates at 1.5 × 10^4^ cells per well. The cells were divided into a nontransfection control group and an NC-shRNA lentivirus transfection group. After 24 h, the NC-shRNA lentivirus transfection group was incubated in antibiotic-free medium with NC-shRNA lentivirus at MOI values of 2, 5, 10, 20, 50, 100, and 200, along with 6 μg/mL polybrene. All experiments were performed in duplicate. After 8 h of incubation, the culture medium was replaced with fresh complete medium supplemented with antibiotics. Transfection efficiency was assessed by observing GFP fluorescence 24 h after transfection.

### 2.3 Cell viability assay

To verify whether lentiviral transfection has cytotoxic effects, the CCK-8 assay was employed to assess cell viability after the lentiviral transfection. Cells were plated in 6-well plates at a density of 3 × 10^5^ cells per well and divided into four groups: Ctrl (untreated control), NC (NC-shRNA transfection), F (*FCGBP*-shRNA-i2 transfection), and blank (blank control). After 10 h, the NC and F groups were transfected with the corresponding lentivirus at an MOI of 200. Following a 10 h incubation, the lentivirus-containing medium was removed, and cells were washed once with phosphate-buffered saline (PBS) before adding fresh complete medium. After 24 h, cells were trypsinized and replated into 96-well plates at 1 × 10^5^ cells per well.

After 12 h, the culture medium was replaced with 90 μL of serum-free medium, and 10 μL of CCK-8 reagent (CoWin Biosciences Co., Ltd., China) was added to each well. Absorbance at 450 nm (OD450) was measured using a microplate reader at 2, 4, 6, 24, and 48 h after the addition of CCK-8 reagent. Each experimental group included six replicate wells, and the entire procedure was performed in three independent experiments (*n* = 3).

### 2.4 Optimal lentiviral interference fragment selection

To select higher efficiency interference fragments, we compared the interference efficiency of two different lentiviral interference fragments. Cells were plated into 24-well plates at a density of 5.5 × 10^4^ cells per well and assigned to the following groups (with two replicates per group): control (no transfection), F1 (transfected with *FCGBP*-shRNA-i1), and F2 (transfected with *FCGBP*-shRNA-i2). At 24 h after seeding, the F1 and F2 groups were transfected with their respective lentiviruses at MOI 100. To enhance transfection efficiency, the viral supernatant was supplemented with 3 μL of polybrene (6 μg/mL). After 8 h of incubation, the viral supernatant was removed, and the cells were subjected to two gentle washes with PBS before adding fresh complete medium containing 1% penicillin–streptomycin.

Transfection efficiency was preliminarily assessed by observing GFP expression under a fluorescence microscope after 48 h of transfection. Then, RNA was extracted from the cells using the TRIzol method (1 mL per well), and the levels of *FCGBP* mRNA expression were quantified by qRT-PCR to identify the most interference-efficient shRNA fragment.

### 2.5 Western blotting analysis

To further evaluate the efficiency of FCGBP protein interference, we assessed the expression levels of FCGBP protein via Western blotting. Cells were plated in 6-well plates at a density of 3 × 10^5^ and assigned to Ctrl (control) and F (*FCGBP*-i2-shRNA transfection) groups. After 10 h of lentiviral transfection, the medium was replaced with fresh complete medium. Following 48 h, cells were lysed using IP lysis buffer (Servicebio Technology Co., Ltd., China). Supernatants were collected after centrifugation, supplemented with 5× SDS-PAGE Protein Loading Buffer (Servicebio Technology Co., Ltd., China), and boiled in a metal bath at 96 °C for 10 min. Samples were separated on a 15% Bis–Tris gel and transferred to a polyvinylidene difluoride membrane. Western blotting was performed using primary antibodies against FCGBP (Cloud-Clone PAP389Hu01) and GAPDH (Proteintech 1E6D9). After incubation with the appropriate secondary antibodies, protein bands were visualized using the Special Supersensitive ECL Chemiluminescence Kit (Beyotime P0018AS). Each group included three technical replicates.

### 2.6 Optimal multiplicity of infection for *P. multocida* infection cell model

To select the optimal infection MOI and the optimal infection time, we conducted a selection of infection conditions. Cells were distributed into 24-well plates at a density of 5 × 10^4^ cells per well. After 24 h of incubation, the culture medium was changed to antibiotic-free medium. Based on the preliminary work conducted in our laboratory, we selected the optimal infection conditions by testing a range of MOIs (10, 50, 100, and 200) for *P. multocida*. With two replicate wells per MOI, followed by co-incubation. Cellular morphology was monitored at 2, 4, and 6 h post-infection (hpi) to define the optimal conditions for infection, i.e., the minimal MOI and earliest time point inducing a significant CPE.

### 2.7 *Pasteurella multocida* infection experiment and sample collection

After determining the optimal infection conditions, we conducted a full-scale infection experiment. Cells were plated into 6-well plates at a density of 3 × 10^5^ cells per well and assigned to the following six experimental groups (with three replicates per group): Ctrl (untreated control), CP (*P. multocida* infection), NC (NC-shRNA transfection), NCP (NC-shRNA transfection + *P. multocida* infection), F (*FCGBP*-shRNA-i2 transfection), and FP (*FCGBP*-shRNA-i2 transfection + *P. multocida* infection). The CP, NCP, and FP groups were infected with *P. multocida* at an MOI of 100 for 4 h following a 24 h period. After infection, the supernatant from each well was collected for subsequent analysis via ELISA. The cells were then washed three times with PBS and lysed directly in the wells using TRIzol reagent. The resulting lysates were snap-frozen in liquid nitrogen and maintained at −80 °C until subsequent RNA extraction.

### 2.8 mRNA library construction and RNA-seq

Each experimental group included three technical repetitions. A total of 18 cell samples from different treatment groups were sent to Guangzhou GENEDENOVO Biotechnology Co., Ltd. (Guangzhou, China) for RNA sequencing. Total RNA was isolated using TRIzol reagent. After extraction, RNA integrity was assessed by agarose gel electrophoresis and using an Agilent 2100 bioanalyzer (Agilent Technologies, Santa Clara, CA, USA). The Hieff NGS® Ultima Dual-mode mRNA Library Prep Kit was employed to prepare sequencing libraries, which were then subjected to quality control using the DNA 1000 Assay Kit (Agilent Technologies, 5067-1504, USA). The libraries with sufficient quality were subsequently sequenced on an Illumina NovaSeq X Plus platform for transcriptome analysis.

### 2.9 Bioinformatics analysis

Following RNA-seq, the raw data were subjected to quality control and filtered using fastp software.^1^ The filtered reads were then mapped to the reference genome in HISAT2 (17). Gene expression levels were estimated using RSEM (18), and differential expression analysis was performed using DESeq2 (19). The OmicShare tool suite^2^ was used for data visualization and functional analyses, which specifically included principal component analysis (PCA), heatmap generation, and Gene Ontology (GO) and Kyoto Encyclopedia of Genes and Genomes (KEGG) enrichment analyses. Finally, protein–protein interaction (PPI) networks were generated using the STRING database^3^ and visualized in Cytoscape (v3.10.2).

### 2.10 Enzyme-linked immunosorbent assay

To validate the dysregulated immune pathways identified by RNA-seq, the levels of secreted cytokines. The levels of key proinflammatory cytokines (IFN-α, TNF-α, CCL5, IL-17, and IL-6) were measured by ELISA kits (Xiamen Lun Changshuo Biotechnology Co., Ltd., China) according to the manufacturer’s instructions. Optical density was promptly measured at 450 nm for each well to calculate sample concentrations.

### 2.11 Quantitative real-time PCR validation

To validate the results of RNA-seq, genes *ISG15*, *RSAD2*, *TRANK1*, *IFIH1*, *DDX58*, *IMP3*, *RRP9*, *FADD*, *PIK3CA*, and *COL1A2* were selected for analysis via qRT-PCR. Total RNA was first reverse-transcribed into cDNA using the 5× FastKing-RT SuperMix (Tiangen Biotech, Beijing, China). Then, qRT-PCR was conducted on a Langji Q2000B real-time system (Langji, Hangzhou, China) using the SYBR Green Pro Taq HS Premixed qPCR Kit (Hunan Aikrui Bioengineering, China). Gene-specific primers (Supplementary Table S1) were designed using the NCBI Primer-BLAST tool.^4^ The gene encoding glyceraldehyde-3-phosphate dehydrogenase (*GAPDH*) was used as an internal reference, with relative gene expression determined via the comparative 2^−ΔΔCT^ method. The data obtained from qRT-PCR are presented as mean ± standard deviation (SD) of three independent biological replicates, each biological replicates measured in 3 technical replicates.

### 2.12 Statistical analysis

Data are presented as mean ± SD from at least three biological replicates (*n* = 3). Differences between groups were analyzed using one-way ANOVA followed by multiple comparisons tests in GraphPad Prism (v10.1.2). Statistical significance was set at *p* < 0.05.

## 3 Results

### 3.1 Optimal multiplicity of infection for lentiviral transduction and cell viability assay

**Figure 1:**
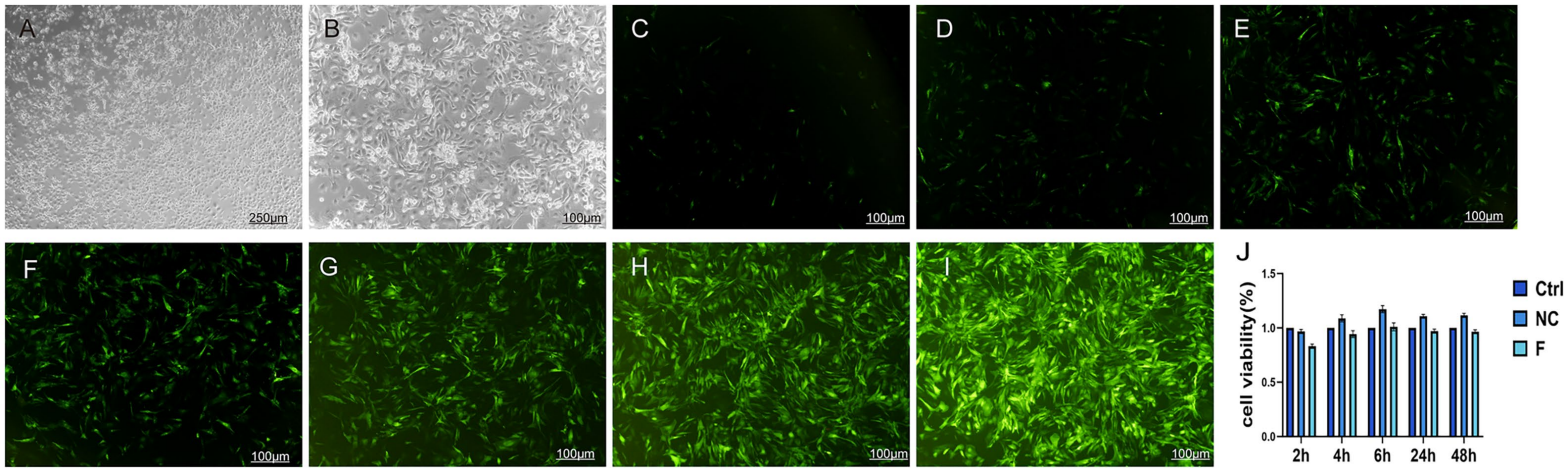
Optimal multiplicity of infection for lentiviral transduction and CCK-8 cell activity detection. **(A)** Bright-field images of goat bronchial epithelial cells under a 4× inverted microscope. **(B)** Bright-field images of goat bronchial epithelial cells under a 10× inverted microscope. **(C–I)** Fluorescence efficiency of cells transfected with NC-shRNA measured at MOI values of 2, 5, 10, 20, 50, 100, and 200. **(J)** CCK-8 assay to detect the effect of lentiviral transfection on cell viability. Data are presented as mean ± SD (*n* = 3).

Goat bronchial epithelial cells exhibited an irregular, cobblestone-like shape under bright-field microscopy (Figures 1A,B). To establish optimal conditions for transfection, cells were transfected with lentiviral particles at MOI values of 2, 5, 10, 20, 50, 100, and 200. Fluorescence imaging at 24 h post-transfection (Figures 1C–I) demonstrated a positive correlation between MOI and transfection efficiency. To ensure that subsequent phenotypic observations were not influenced by cytotoxicity, we first evaluated whether the cell viability was affected by lentiviral transfection. We assessed the impact of lentiviral transfection on cell viability using CCK-8 assays. Results showed that at 48 h, the NC group exhibited a cell viability rate of 111%, while the F group demonstrated a 97% cell viability rate (Figure 1J). No significant cytotoxicity or morphological changes were induced by the lentivirus across the MOI range tested. As the maximal fluorescence efficiency was observed at MOI 200, this value was selected for all subsequent experiments.

### 3.2 Optimal lentiviral interference fragments and knockdown efficiency assessment

**Figure 2:**
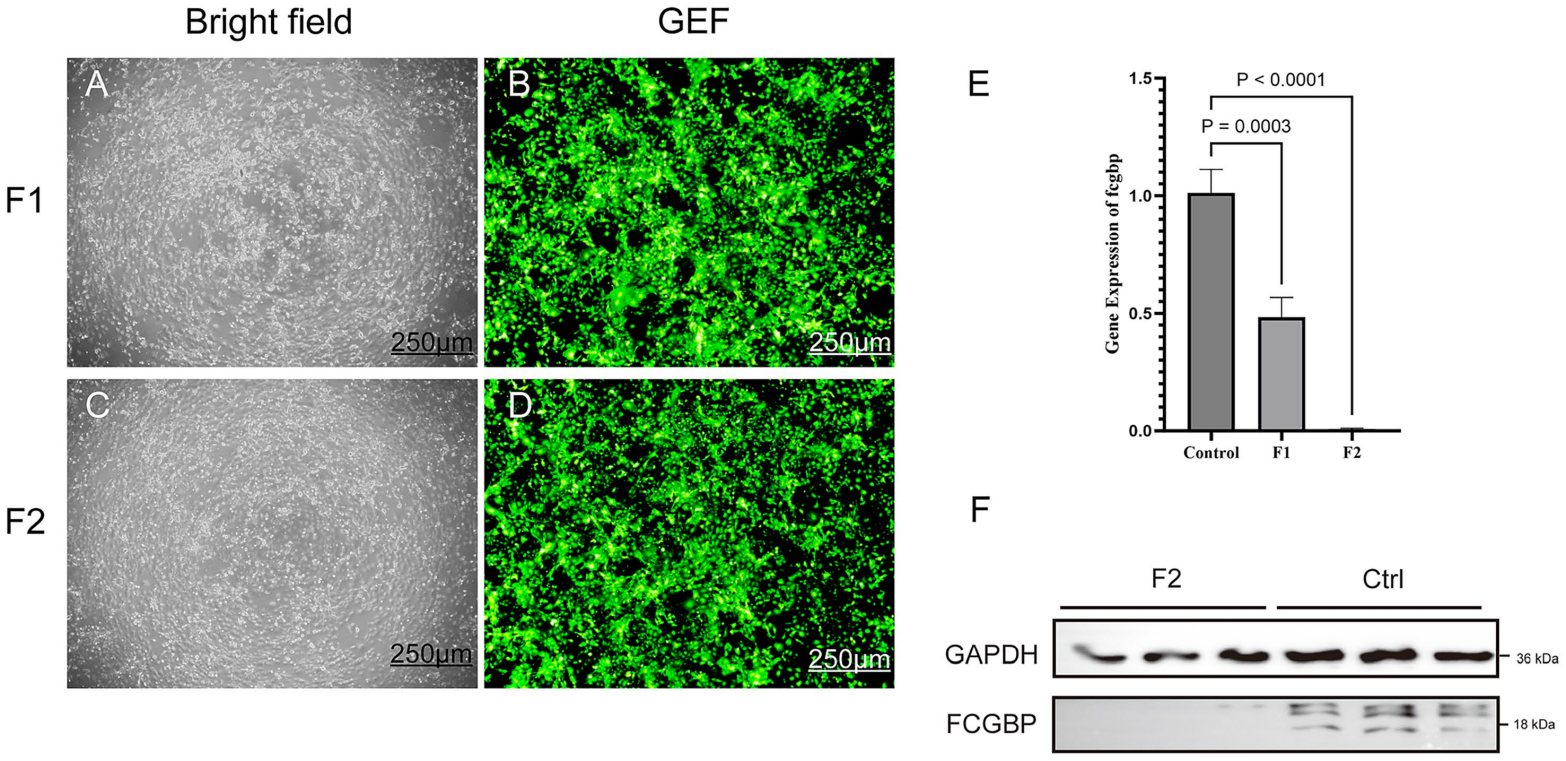
Evaluation of *FCGBP* knockdown efficiency. **(A,B)** Bright-field and corresponding fluorescence images of cells transduced with *FCGBP*-shRNA-i1 (MOI 200). **(C,D)** Bright-field and fluorescence images of cells transduced with *FCGBP*-shRNA-i2 (MOI 200). Scale bars in **(A–D)**: 250 μm. **(E)** qRT-PCR results demonstrating the interference efficiency of *FCGBP*-shRNA-i1 (*p* = 0.0003) and *FCGBP*-shRNA-i2 (*p* < 0.0001). **(F)** Western blotting results demonstrated the efficiency of *FCGBP* knockdown. GAPDH served as the loading control.

To identify the most effective *FCGBP*-targeting shRNA fragment, goat bronchial epithelial cells were transfected at MOI 200 with one of two distinct lentiviral constructs: group F1 (*FCGBP*-shRNA-i1) and group F2 (*FCGBP*-shRNA-i2). As shown in Figures 2A–D, qRT-PCR analysis confirmed that *FCGBP*-shRNA-i2 achieved a superior knockdown efficiency compared to *FCGBP*-shRNA-i1. Therefore, the *FCGBP*-shRNA-i2 construct at MOI 200 was selected for all subsequent experiments (Figure 2E). Western blotting analysis showed that FCGBP expression was significantly reduced in the F2 (*FCGBP*-i2-shRNA) group compared with that in the Ctrl group (Figure 2F).

### 3.3 Establishment of a *P. multocida* infection goat bronchial epithelial cell model

**Figure 3:**
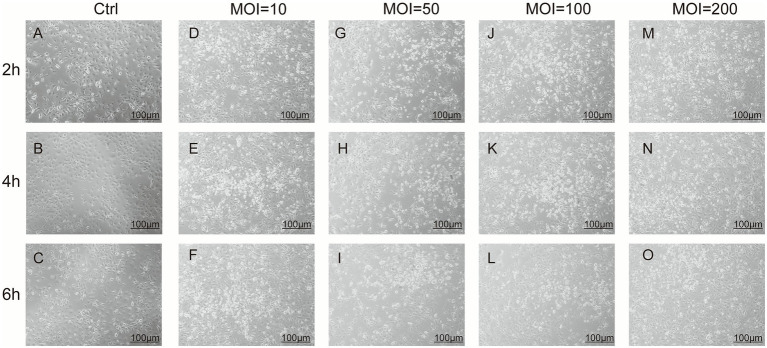
Morphological changes in goat bronchial epithelial cells following *P. multocida* infection. **(A–O)** Images of goat bronchial epithelial cells infected with *P. multocida* at MOI values of 0, 10, 50, 100, and 200 at 2–6 h post-infection.

The goat bronchial epithelial cells in the Ctrl group maintained an irregular cobblestone-like shape throughout the observation period (6 h) (Figures 3A–C). The changes in cell morphology following infection with *P. multocida* at various MOIs (10, 50, 100, and 200) over time are summarized in Figures 3D–O. The onset of cell death was observed across all infected groups as early as 2 hpi, though without notable morphological changes, and within 4 hpi, cell death became more pronounced. Concurrently, cells in the group at 200 MOI began to exhibit a slightly round shape. This progressive rounding was evident in both the MOI 100 and MOI 200 groups by 6 hpi. Based on these observations, a robust infection model at MOI 100 with an infection duration of 6 h was established for the subsequent experiments involving the CP, NCP, and FP groups.

### 3.4 Quality control analysis of RNA-seq data

Quality control and filtering of the raw RNA-seq reads in fastp software involved removing reads containing adapters, those with >10% N bases, low-quality reads (where >50% of bases had a Phred score ≤20), and poly-A reads (Supplementary Table S2). The clean reads were then aligned to the reference genome (Assembly GCA_001704415.2), achieving a high mapping rate of over 93% across all samples (Supplementary Table S3).

**Figure 4:**
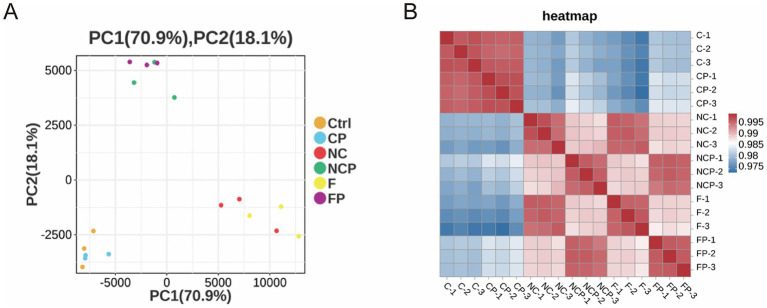
Transcriptome data from different experimental groups yield distinct clusters. **(A)** Principal component analysis (PCA) plot showing distinct clustering of samples based on treatment. **(B)** Correlation heatmap of gene expression profiles. Ctrl, untreated control; CP, *P. multocida* infection; NC, NC-shRNA transfection; NCP, NC-shRNA transfection + *P. multocida* infection; F, *FCGBP*-shRNA-i2 transfection; FP, *FCGBP*-shRNA-i2 transfection + *P. multocida* infection.

Using the high-quality sequencing data, we first evaluated the overall transcriptomic differences among the samples. PCA of the transcriptomic data revealed a clear segregation of the experimental groups (Figure 4A). The first two principal components (PC1 and PC2) explained 70.9 and 18.1% of the total variance, respectively. Notably, the Ctrl and CP groups formed distinct clusters, separate from the NC and F groups as well as from the FP and NCP groups.

The correlation heatmap generated for gene expression profiles further supported these findings (Figure 4B). As expected, biological replicates within the same group clustered together, demonstrating high correlation. In contrast, the Ctrl and CP groups showed low levels of correlation with the other treatment groups.

### 3.5 FCGBP knockdown amplifies transcriptional changes upon *P. multocida* infection

**Figure 5:**
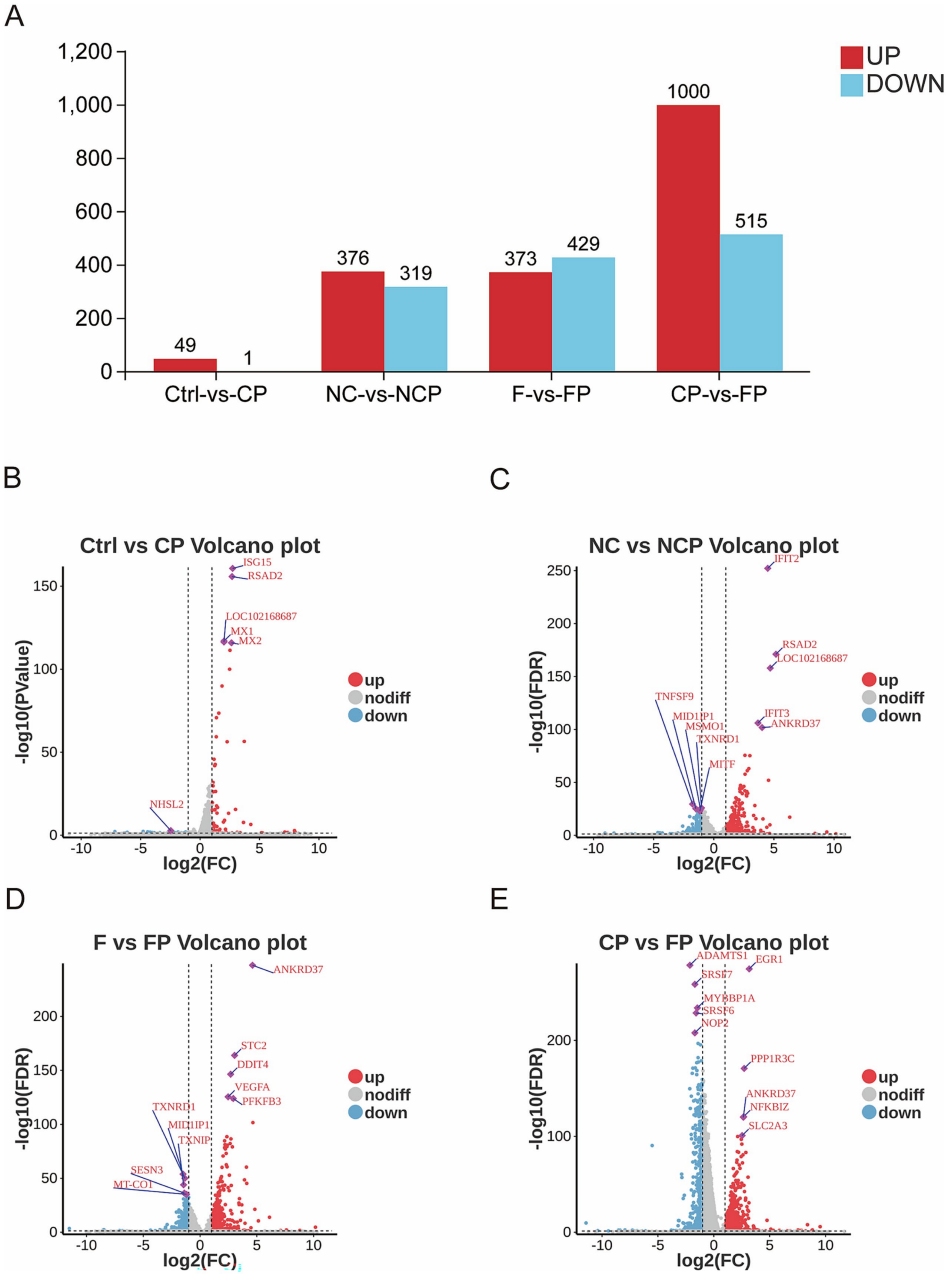
Bar charts and volcano plots of differentially expressed genes across experimental groups. **(A)** Bar plot summarizing the number of differentially expressed genes (DEGs) (|log_2_FC| > 1, FDR < 0.05) for key comparisons. **(B–E)** Volcano plots of DEGs in Ctrl vs. CP **(B)**, NC vs. NCP **(C)**, F vs. FP **(D)**, and CP vs. FP **(E)**. The red dots illustrate up-regulated genes, the blue dots represent downregulated genes, and the grey dots show insignificant genes. The top five significantly differentially expressed genes (DEGs) are marked in the figure. Ctrl, untreated control; CP, *P. multocida* infection; NC, NC-shRNA transfection; NCP, NC-shRNA transfection + *P. multocida* infection; F, *FCGBP*-shRNA-i2 transfection; FP, *FCGBP*-shRNA-i2 transfection + *P. multocida* infection.

Following quality control, DEGs were identified from the RNA-seq data. For the NCP versus FP comparison, significance was defined as *p* < 0.05 and |log2FC| > 1, whereas for all other comparisons, an FDR < 0.05 and |log2FC| > 1 were applied. Comparative transcriptomic analysis revealed a profound impact of FCGBP knockdown on the host response to infection. Infection alone induced 50 DEGs for Ctrl versus CP (49 upregulated and 1 downregulated) and 695 DEGs for NC versus NCP (376 upregulated and 319 downregulated), FCGBP-knockdown cells exhibited a dramatically amplified response with 802 DEGs for F versus FP (373 upregulated and 429 downregulated), 1,515 DEGs for CP versus FP (1,000 upregulated and 515 downregulated) (Figure 5A), and 70 DEGs for NCP versus FP (47 upregulated and 23 downregulated) (Supplementary Figure S1A), with the CP versus FP group comparison exhibiting the most substantial transcriptional alterations. We annotated the top five significantly upregulated and downregulated DEGs for each comparison group (Figures 5B–E, Supplementary Figure S1B).

### 3.6 FCGBP modulates pathways and immune-related DEGs in goat bronchial epithelial cells infected with *P. multocida*

**Figure 6:**
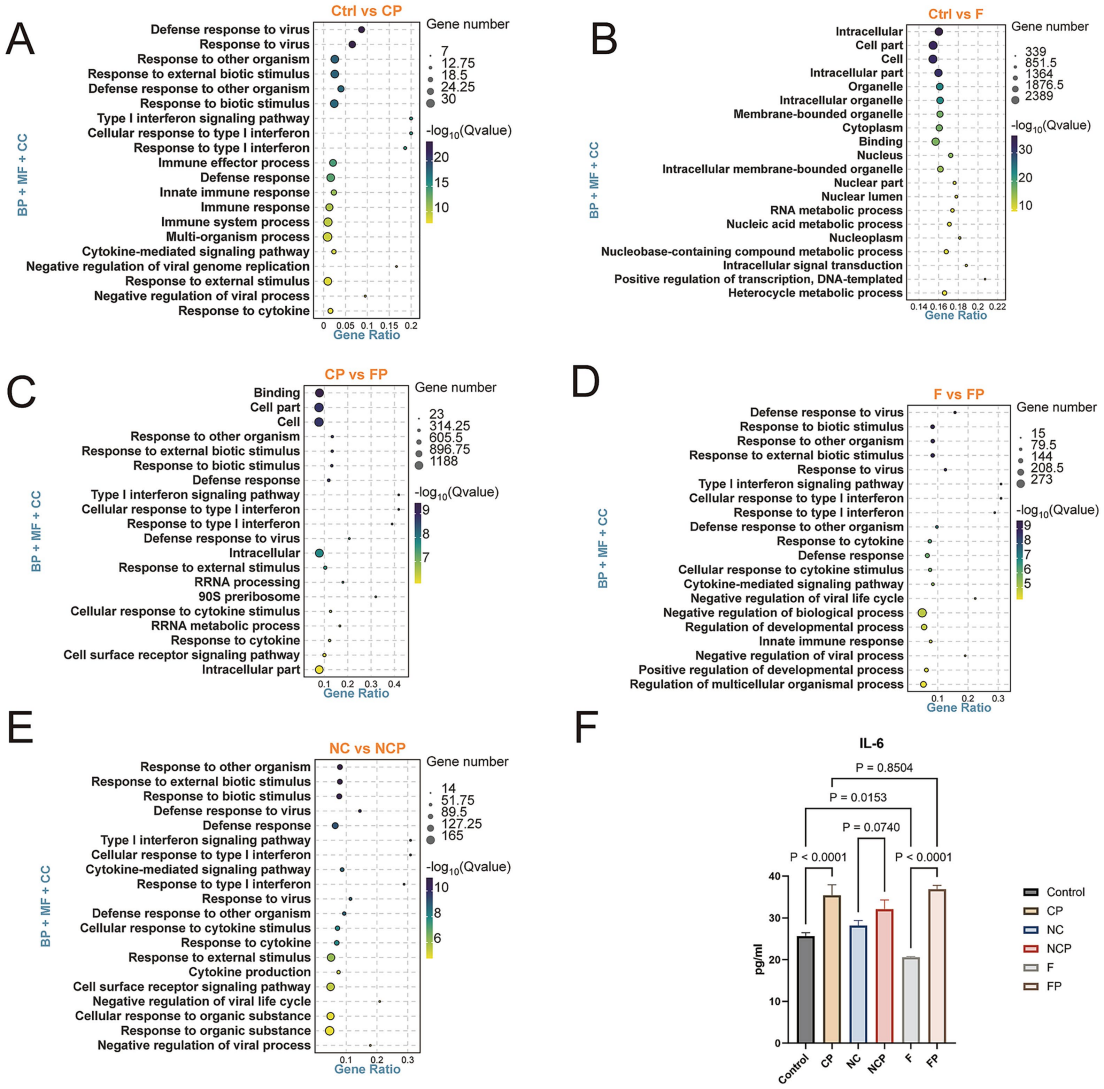
Gene ontology (GO) enrichment analysis of DEGs top 20 enriched terms (by smallest *Q*-value) are shown for comparisons **(A)** Ctrl vs. CP, **(B)** Ctrl vs. F, **(C)** CP vs. FP, **(D)** F vs. FP, and **(E)** NC vs. NCP. Based on functional classification, these DEGs fall into three categories: biological process (BP), cellular component (CC), and molecular function (MF). The size of each circle in the figure represents the number of genes. **(F)** ELISA assay for IL-6 expression levels. Data are mean ± SD (*n* = 3). Ctrl, untreated control; CP, *P. multocida* infection; NC, NC-shRNA transfection; NCP, NC-shRNA transfection + *P. multocida* infection; F, *FCGBP*-shRNA-i2 transfection; FP, *FCGBP*-shRNA-i2 transfection + *P. multocida* infection.

To functionally characterize the DEGs, GO enrichment analysis was conducted for the top 20 terms with the smallest *Q*-values (Figure 6). In groups infected with *P*. *multocida* DEGs were enriched in the type I interferon signaling pathway and defense response pathways (Figures 6A,C,D), whereas the Ctrl versus F group showed no enrichment in these immune-related pathways, confirming successful immune activation in goat bronchial epithelial cells upon *P. multocida* infection. We observed from the GO pathway enrichment analysis of the Ctrl versus F comparison group (Figure 6B) that under non-infected conditions, enriched pathways predominantly clustered around Cell part, Cell, and Organelle pathways. This indicates that under normal physiological conditions, the primary function of the FCGBP is associated with maintaining the fundamental structural integrity of cells. Interestingly, we observed, in the comparison between the Ctrl and F groups at the transcriptomic level, that *IL-6* gene expression was significantly downregulated, indicating that FCGBP deficiency leads to reduced expression of certain fundamental immune factors. This finding was confirmed by ELISA (Figure 6F, *p* = 0.0153), indicating that FCGBP deficiency compromises baseline immune signaling even in the absence of pathogen challenge. Further supporting its role in structural integrity, in the Ctrl vs. F group comparison, DEGs were also associated with pathways like “biological adhesion” and “cell junction” (Supplementary Figure S3), reinforcing the notion that FCGBP contributes to epithelial barrier maintenance. In infected cells, the CP versus FP comparison revealed DEGs enriched in “cell surface receptor signaling”, “response to lipopolysaccharide,” and “response to molecules of bacterial origin” (Figure 6D). Similarly, NCP versus FP comparisons showed DEGs enriched in the cell periphery and plasma membrane, further suggesting that FCGBP maintains the structural integrity of the cell membrane during infection (Supplementary Figure S2). This pivotal finding indicated that the FCGBP is closely related to the cell barrier and cell surface receptor signaling pathways and directly participates in the host cell’s perception and defense response against bacteria.

**Figure 7:**
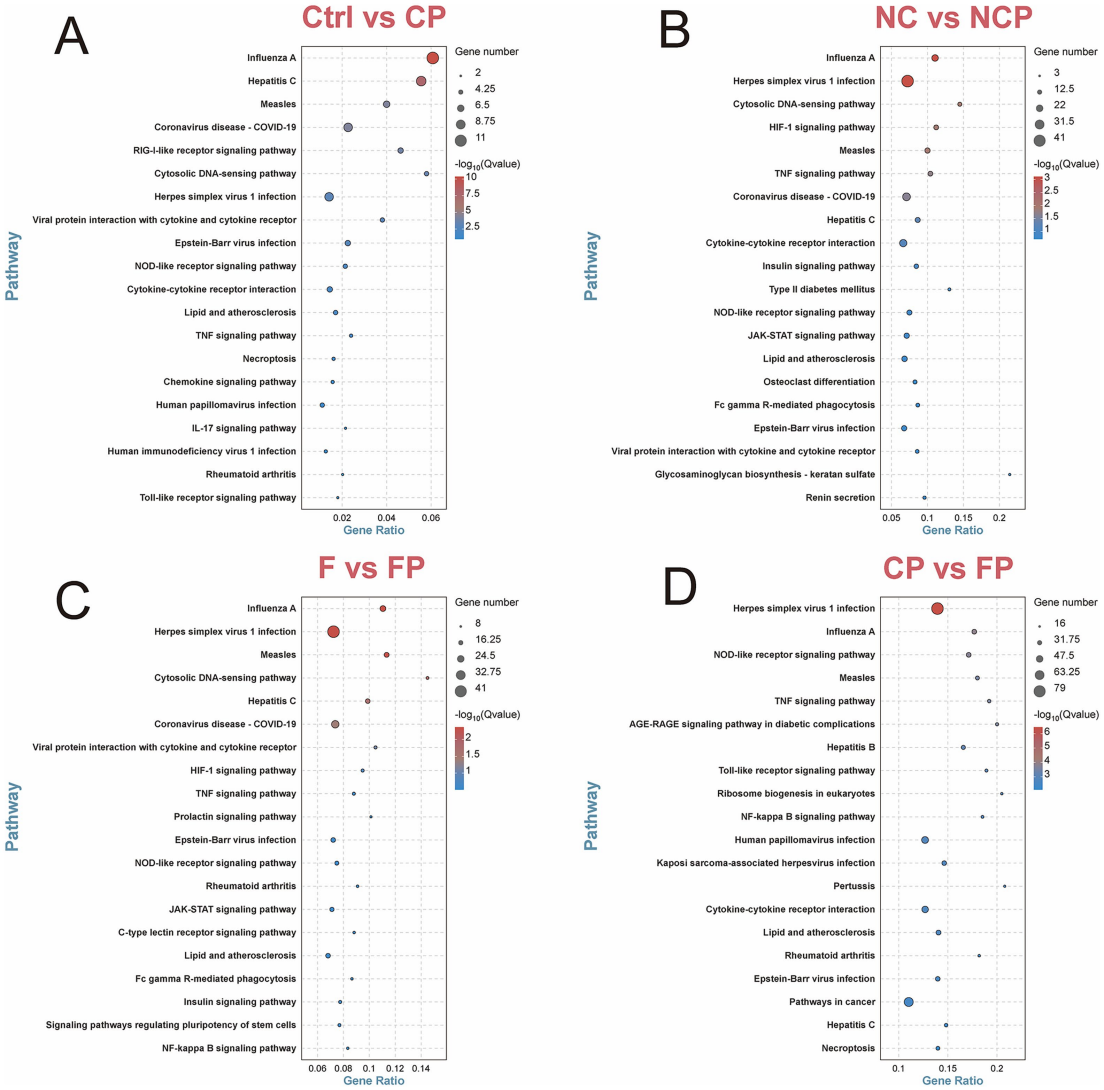
KEGG pathway enrichment analysis of DEGs. Top 20 enriched pathways (by smallest *Q*-value) for comparisons **(A)** Ctrl vs. CP, **(B)** NC vs. NCP, **(C)** F vs. FP, and **(D)** CP vs. FP. The circle size represents the gene number. Ctrl, untreated control; CP, *P. multocida* infection; NC, NC-shRNA transfection; NCP, NC-shRNA transfection + *P. multocida* infection; F, *FCGBP*-shRNA-i2 transfection; FP, *FCGBP*-shRNA-i2 transfection + *P. multocida* infection.

**Figure 8:**
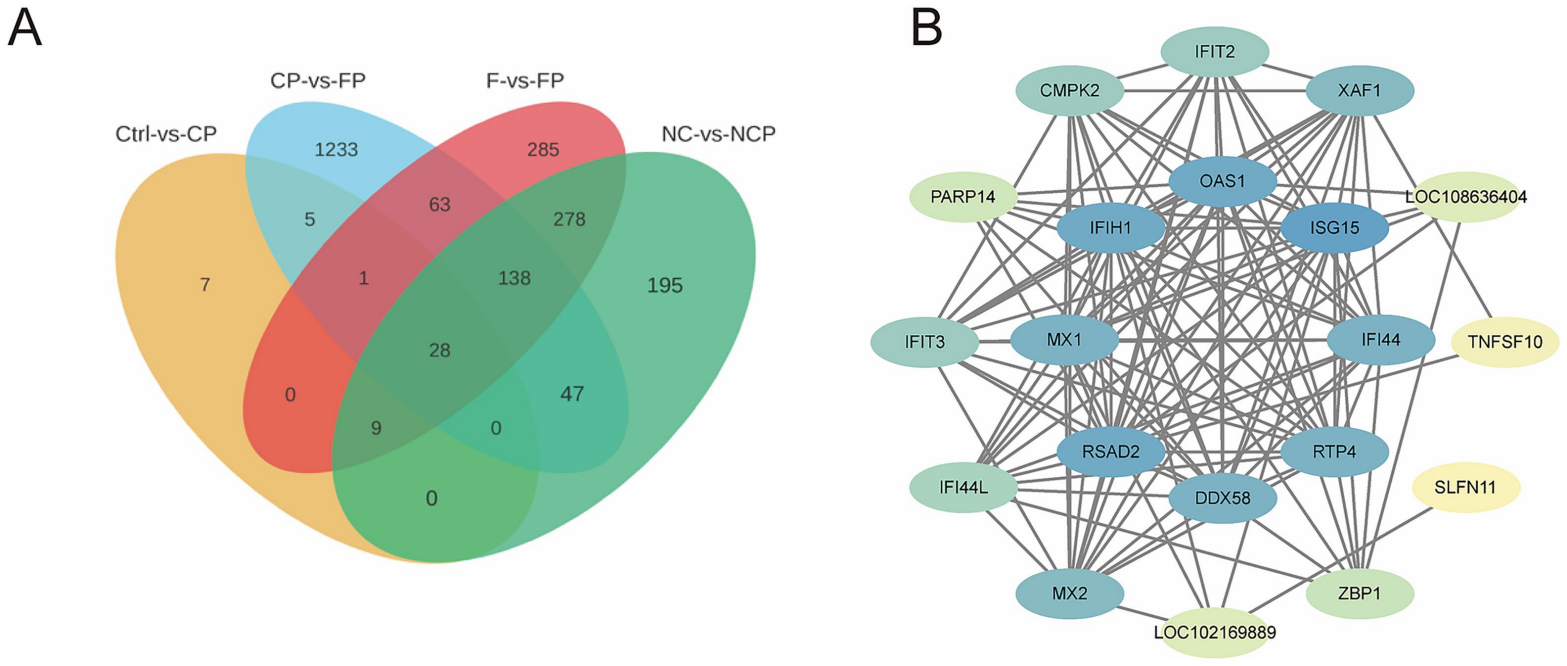
Common DEGs and their protein interaction network. **(A)** Venn diagram showing the overlap of DEGs across four key comparisons: Ctrl vs. CP (orange), CP vs. FP (blue), F vs. FP (red), and NC vs. NCP (green). 28 genes are common to all comparisons. **(B)** Protein–protein interaction network for the 28 DEGs overlapping across the four group comparisons shown in the Venn diagram in part A. Node size corresponds to the degree of connection. Ctrl, untreated control; CP, *P. multocida* infection; NC, NC-shRNA transfection; NCP, NC-shRNA transfection + *P. multocida* infection; F, *FCGBP*-shRNA-i2 transfection; FP, *FCGBP*-shRNA-i2 transfection + *P. multocida* infection.

This role was further confirmed by KEGG pathway analysis (top 20 pathways based on the smallest *Q*-value; Figure 7). While the DEGs identified in the Ctrl versus CP group comparison were enriched in core inflammatory pathways (e.g., NLR, TNF, and cytokine–cytokine receptor interaction pathways; Figure 7A), those detected in both the NC versus NCP and F versus FP group comparisons showed additional enrichment in the C-type lectin receptor signaling pathway and Fc gamma R-mediated phagocytosis (Figures 7B,C), underscoring the broad involvement of the FCGBP in pulmonary immunity. Most significantly, the CP versus FP group comparison exhibited the most pronounced inflammatory signature, with DEGs highly enriched in the NLR, TNF, TLR, NF-kappa B, and cytokine–cytokine receptor interaction pathways (Figure 7D). This demonstrated that FCGBP deficiency exacerbates the inflammatory response to *P*. *multocida* infection. To identify a consensus immune signature, the overlap of DEGs across the four group comparisons was then analyzed, revealing 28 common DEGs, as shown in the Venn diagram in Figure 8A. PPI network analysis of these genes highlighted a core set of hub genes, with the top 10 ordered as follows based on the degree value: *ISG15, OAS1, IFIH1, RSAD2, MX1, IFI44, DDX58, RTP4, MX2, XAF1*, and *CMPK2* (Figure 8B). The central position of these genes in the network suggests they play an essential regulatory role in the host response to *P. multocida* infection.

### 3.7 PPI networks identify core interferon-stimulated genes and disrupted adhesion modules

**Figure 9:**
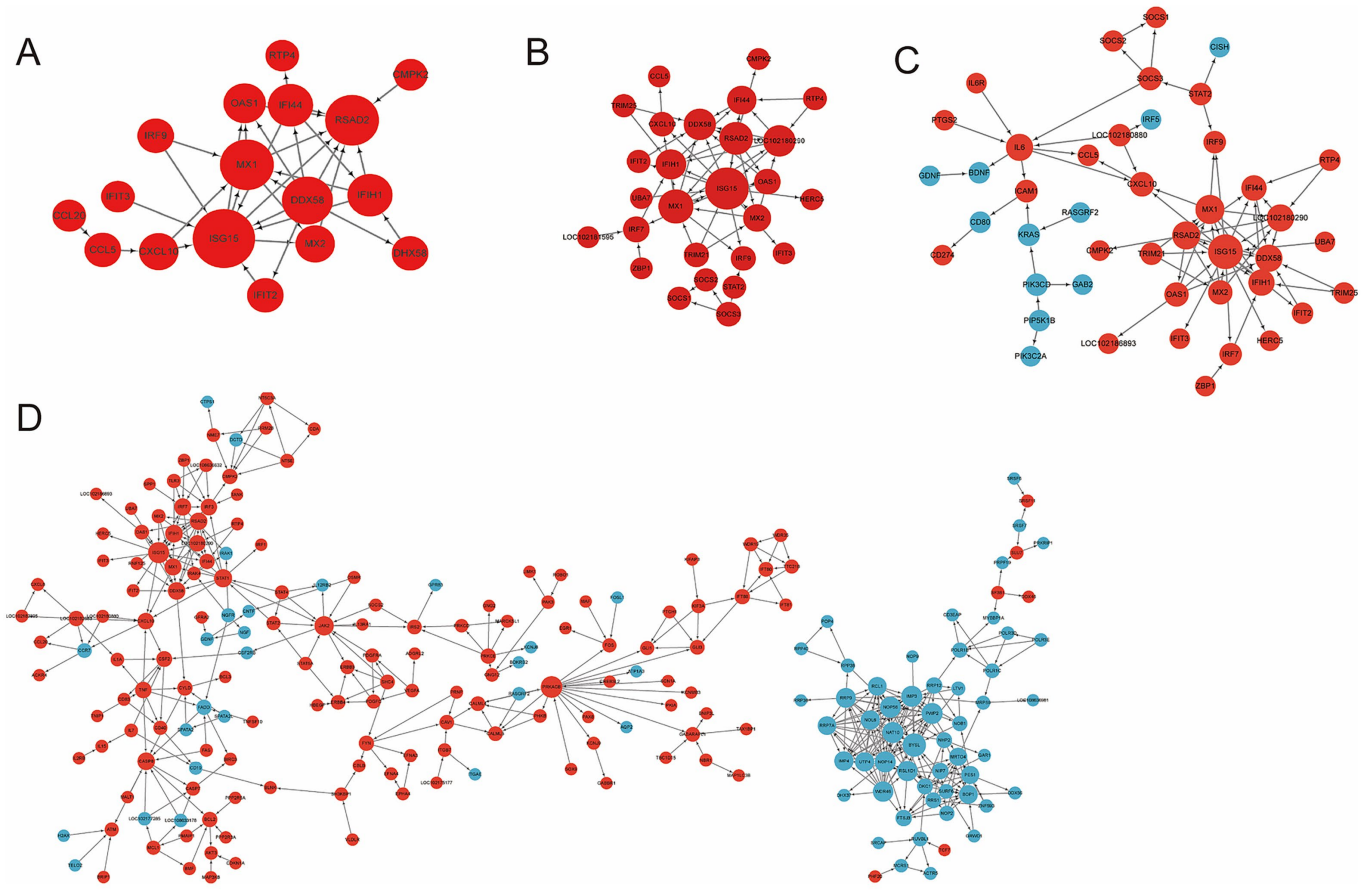
Protein–protein interaction network mapping for differentially expressed genes. Networks for DEGs from comparisons **(A)** Ctrl vs. CP, **(B)** NC vs. NCP, **(C)** F vs. FP, and **(D)** CP vs. FP. Nodes represent proteins (red: upregulated, blue: downregulated), node size reflects the degree value, plotted with high confidence.

To further elucidate the functional relationships among dysregulated genes identified in the preceding pathway analyses, protein–protein interaction (PPI) networks were constructed. PPI analysis reinforced the transcriptomic findings, revealing two major dysregulated modules in *FCGBP*-deficient infected cells (Figure 9). First, a core cluster of interferon-stimulated genes (e.g., *ISG15, MX1, RSAD2, OAS1*, and *IFIH1*) was hyperactivated, quantitatively confirming the amplified interferon response. Concurrently, a module comprising genes essential for cell adhesion and junction integrity (e.g., *ITGAE, NGFR, GDNF* and *AQP2*) was suppressed. These findings indicate that FCGBP deficiency may compromise barrier integrity and disrupt fundamental cellular processes, potentially leading to a stressed and dysfunctional state in infected cells.

### 3.8 FCGBP knockdown regulates cytokine secretion

**Figure 10:**
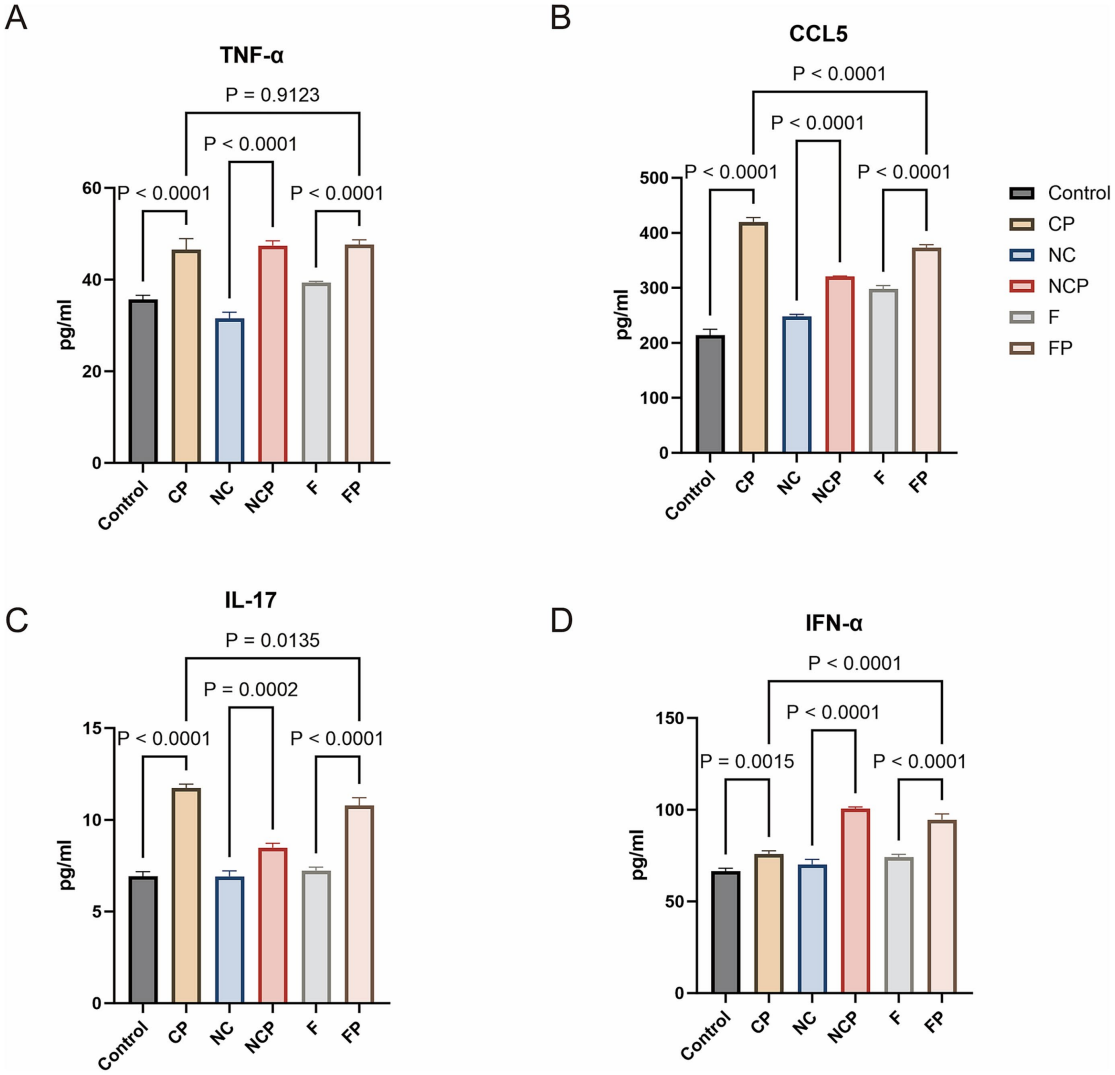
*FCGBP* knockdown differentially regulates cytokine secretion during infection. **(A–D)** Results of ELISA measuring the concentrations of key inflammatory cytokines, i.e., TNF-α, CCL5, IL-17, and IFN-α. The experiment employed one-way analysis of variance and Tukey’s multiple comparison test to assess the statistical significance of the mean differences between different experimental groups and the control group. Data are presented as the mean ± standard deviation (SD) from three biologically independent experiments (*n* = 3). Statistical significance was set at *p* < 0.05. Ctrl, untreated control; CP, *P. multocida* infection; NC, NC-shRNA transfection; NCP, NC-shRNA transfection + *P. multocida* infection; F, *FCGBP*-shRNA-i2 transfection; FP, *FCGBP*-shRNA-i2 transfection + *P. multocida* infection.

To validate the transcriptional changes at the protein level, we quantified the concentrations of secreted cytokines. Quantification of the concentrations of IFN-α, TNF-α, CCL5, IL-17 across different treatment groups via ELISA revealed that the levels of all five cytokines in the CP, NCP, and FP groups were significantly upregulated compared to those in their respective uninfected controls (Ctrl, NC, and F groups). Notably, FCGBP knockdown exerted distinct, cytokine-specific effects. Compared to the CP group, the FP group exhibited significantly lower levels of IL-17 and CCL5, whereas IFN-α concentration was markedly elevated (Figure 10). These protein levels are fully consistent with the transcriptomic profiles.

### 3.9 qRT-PCR validation of key hub genes confirms transcriptomic data accuracy

**Figure 11:**
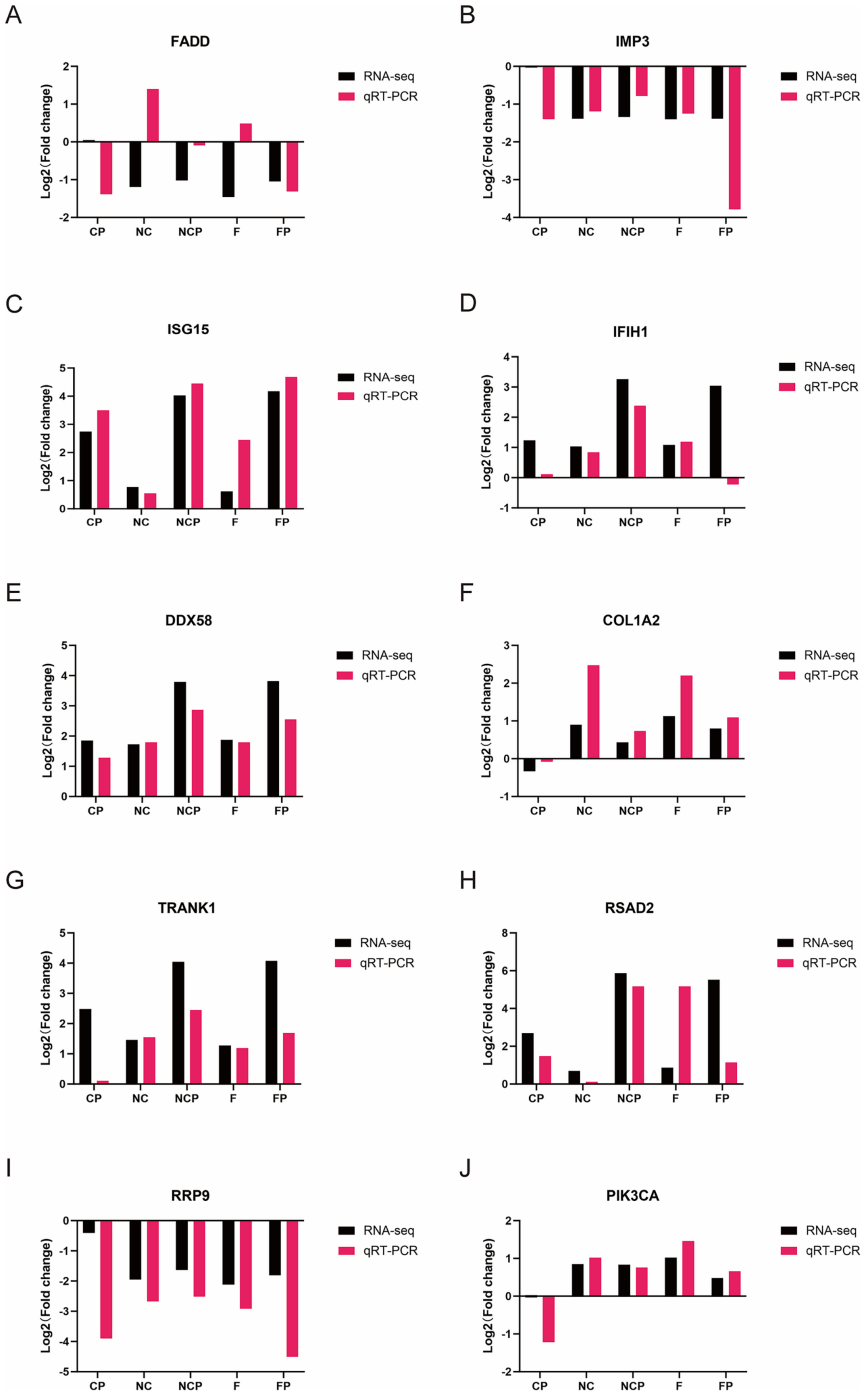
qRT-PCR validation of key DEGs identified by RNA-seq **(A–J)**. Comparison of the expression levels of *FADD, IMP3, ISG15, IFIH1, DDX58, COL1A2, TRANK1, RSAD2, RRP9*, and *PIK3CA* obtained by qRT-PCR and RNA-seq. Data are mean ± SD (*n* = 3). Ctrl, untreated control; CP, *P. multocida* infection; NC, NC-shRNA transfection; NCP, NC-shRNA transfection + *P. multocida* infection; F, *FCGBP*-shRNA-i2 transfection; FP, *FCGBP*-shRNA-i2 transfection + *P. multocida* infection.

To experimentally validate the transcriptomic data, the expression levels of the 10 above-mentioned hub genes consistently identified in the PPI networks were quantified by qRT-PCR. As shown in Figure 11, a strong concordance between the results of RNA-seq and those of qRT-PCR was observed for 9 out of 10 genes (*ISG15, RSAD2, TRANK1, IFIH1, DDX58, IMP3, RRP9, PIK3CA,* and *COL1A2*), thereby confirming the overall accuracy of our sequencing analysis. An exception was the *FADD* gene, which showed a divergent expression pattern. This discrepancy is likely attributable to differences in transcript variants or post-translational modifications, which can lead to inconsistent results between RNA-seq and qRT-PCR assays.

## 4 Discussion

Our results demonstrate that FCGBP deficiency markedly exacerbates the inflammatory response induced by *P. multocida* infection and modulates the activity of key immune signaling pathways. These findings underscore the critical regulatory role of FCGBP in mucosal immunity within the goat respiratory tract. Transcriptional changes were primarily observed in specific antibacterial and inflammatory pathways, as confirmed by RT-qPCR and ELISA results.

To clarify the direct effects of *P. multocida* infection, differential gene expression was analyzed between the Ctrl and CP groups. The results revealed a significant enrichment of DEGs in the type I interferon signaling pathway, consistent with a previous study by Wu et al. (15), which reported GO enrichment in the interferon-β and interferon-γ-mediated signaling pathways. Furthermore, the proinflammatory response observed in our model is consistent with previously published *in vivo* data. Specifically, in a comparison between 36 sheep infected with *P. multocida* and 20 healthy control sheep, El-Deeb and Elmoslemany (20) demonstrated that the serum levels of the proinflammatory cytokines IL-1α, IL-1β, and IL-6 were significantly elevated in the infected group. The reported increase in IL-6 in particular is consistent with the ELISA results obtained in the present study. Furthermore, our transcriptomic analysis revealed that the DEGs identified in the Ctrl versus CP group comparison were significantly enriched in several innate immune signaling pathways, including the RIG-I-like receptor (RLR), NLR, and TLR pathways. NLRs are cytosolic pattern recognition receptors (PRRs) pivotal to mucosal immune defense (21), whereas TLRs sense pathogen-associated molecular patterns (PAMPs) (22). Specifically, recognition of *P. multocida* lipopolysaccharide by TLRs activates innate defense mechanisms (23), leading to the induction of inflammatory cytokines via downstream signaling cascades (24). Furthermore, a marked induction of genes encoding proteins of the interferon-induced protein with tetratricopeptide repeats family was observed in the CP group but not in the Ctrl group. While typically expressed at low basal levels, pronounced upregulation of these genes in response to infection is associated with the sensing of PAMPs (25). This interpretation is strongly supported by our PPI network analysis of DEGs specific to the CP group. The identified hub genes, which included canonical interferon-stimulated genes (*ISGs*) such as *ISG15, MX1, MX2*, and *OAS1*, as well as genes encoding interferon-induced proteins, like *IFI44*, *IFIT2*, and *IFIT3*, were predominantly associated with the type I interferon signaling pathway. Collectively, these findings demonstrate that *P. multocida* infection effectively engages intracellular PRR pathways, including those mediated by RIG-I and MDA5, triggering a strong ISG response that constitutes the main antibacterial defense in goat epithelial cells. FCGBP knockdown resulted in a significant upregulation of proinflammatory cytokines (e.g., IL-6, IFN-α) secreted by epithelial cells during infection. Moreover, GO enrichment analysis of the NCP versus FP, comparison revealed pathways related to the cell periphery and plasma membrane, as well as molecular functions including actin binding and alpha-actinin binding. This finding supports the hypothesis that FCGBP participates in mucosal immunoregulation, with the main function of maintaining immune homeostasis. Mechanistically, FCGBP likely achieves this through interactions with other glycoproteins (26). The suppression of FCGBP disrupts this homeostatic balance, leading to excessive activation of downstream signaling pathways such as NF-κB upon PAMP recognition, thereby predisposing the host to a dysregulated, hyperinflammatory state. The protective role of FCGBP is further corroborated by clinical and experimental evidence. Huang et al. (27) reported that impaired goblet cell function (including reduced FCGBP) in patients with acute pancreatitis exacerbated proinflammatory cytokine levels in the colon, which is in line with the results obtained in our knockdown model. Notably, in the absence of infection, the F group presented significantly reduced basal IL-6 levels compared to the Ctrl group, a finding consistent with Fu et al. (6), suggesting that FCGBP plays a role in baseline immune tone. However, the critical function of FCGBP becomes most apparent under pathogen infection.

Following FCGBP knockdown and subsequent *P. multocida* infection, the FP group exhibited even higher levels of IL-6 expression than the CP group. This increase was accompanied by a broader and more potent immune activation, including (1) marked upregulation of ISGs, such as *ZBP1, UBA7, HERC5*, and *TRIM25*; (2) evidence of the JAK/STAT signaling pathway involvement (e.g., *STAT2, SOCS1-3* and *IL6R*); and (3) increased expression of the co-inhibitory molecule CD274 (*PD-L1*) (28). This evidence indicates that FCGBP exerts immunoregulatory functions through the NF-κB and JAK/STAT signaling pathways, not only in the ovaries but also in the lungs (6). Specifically, FCGBP knockdown led to the marked upregulation of a broad spectrum of immune-related genes, including inflammatory mediators (e.g., *TNF*, *IL1A*, *IL15*, and *CXCL8*) (29), apoptosis-related molecules (e.g., *CASP8*, *FAS*, and *PMAIP1*) (30), PRRs and adaptors (e.g., *TLR3*, *IRAK1*, and *IRAK4*) (31), key transcription factors (e.g., *STAT1, STAT2, STAT4, IRF1, IRF3,* and *IRF7*) (32), and antigen presentation-related genes (e.g., *CD40* and *CD83*) (33). Concurrently, we observed broad downregulation of genes involved in core cellular functions (such as genes from the *POLR* and *NOP* families) and ribosomal biogenesis. This concerted shift in gene expression, away from homeostasis and toward defense, indicates that FCGBP knockdown drastically rewires the cellular state, heightening pathogen-sensing capabilities, amplifying inflammatory signal transduction, and potentiating immune effector output.

In summary, by integrating the transcriptional changes observed following FCGBP knockdown under both steady-state and infection conditions, we establish that FCGBP is a core regulator of bronchial epithelial homeostasis in goats. Our data provide compelling evidence that FCGBP functions not merely as a responsive element but as a critical proactive guardian. Its suppression compromises epithelial integrity from within, as evidenced by the downregulation of key genes regulating cell adhesion and cilia assembly (e.g., *ITGAE, NGFR, GDNF,* and *AQP2*), which are all associated with epithelial barrier function (34–37), thereby weakening the first line of defense. Following *P. multocida* infection, this is characterized by marked upregulation of proinflammatory cytokines and a robust interferon-stimulated gene profile. This confirms the protective function of this gene in epithelial cells and its role as a molecular buffer that rigidly suppresses ISG pathways and inflammatory signaling, preventing systemic overactivation. Consequently, when FCGBP-deficient cells become infected with *P. multocida*, this pre-compromised epithelial barrier and pre-sensitized immune environment jointly drive a hyperactive pathological response, as detailed in our PPI network analysis. Overall, our findings identify FCGBP as a pivotal core gene for maintaining immune homeostasis in the respiratory epithelium and elucidate the molecular mechanisms through which it regulates airway immunity. They also provide a compelling theoretical foundation for exploring FCGBP-targeted strategies to treat infectious and inflammatory lung diseases.

## 5 Conclusion

This study confirms that FCGBP plays a crucial role in regulating both immune homeostasis and the cellular barrier in goat bronchial epithelial cells. FCGBP deficiency was shown to disrupt this regulatory mechanism, leading to impaired cellular barrier function and severe dysregulation of host immune responses against *P. multocida* infection. Compared to the Ctrl group, DEGs in the F group showed enrichment in the “biological adhesion” and “cell junction” pathways in GO and KEGG analyses. Additionally, PPI network analysis of CP vs. FP revealed downregulation of the *ITGAE, NGFR, GDNF*, and *AQP2* genes, which are associated with cell adhesion, suggesting that FCGBP plays a protective role in maintaining the epithelial cell barrier. Compared to the CP group, DEGs in the FP group were enriched in GO and KEGG pathways, including the type I interferon signaling pathway, defense response, cell surface receptor signaling pathway, response to lipopolysaccharide, and response to molecules of bacterial origin. These findings indicate that, in cells infected with *P. multocida*, FCGBP knockdown compromises the cellular barrier, leading to heightened inflammatory responses and excessive activation of key immune pathways. Furthermore, the analysis of PPI networks revealed that key node genes such as *ISG15, MX1, RSAD2, OAS1*, and *IFIH1* were all associated with type I interferon, highlighting the critical role of the type I interferon pathway in the immune response against bacterial invasion following FCGBP knockdown.

## Data Availability

The datasets presented in this study can be found in online repositories. The names of the repository/repositories and accession number(s) can be found below: https://www.ncbi.nlm.nih.gov/bioproject/PRJNA1347253.

## Footnotes

1
https://github.com/OpenGene/fastp

2
https://www.omicsmart.com

3
https://cn.string-db.org/

4
https://www.ncbi.nlm.nih.gov/
