# Correction: Dysregulated interferon signaling and hyperinflammation upon *FCGBP* knockdown in goat bronchial epithelial cells infected with *Pasteurella multocida*

**DOI:** 10.3389/fvets.2026.1824958

**Published:** 2026-03-23

**Authors:** Yingxue Yang, Xiangyin Chen, Ziying Wang, Lili Wu, Li Du, Hongyan Gao, Si Chen, Qiaoling Chen, Fengyang Wang

**Affiliations:** Hainan Key Laboratory of Tropical Animal Reproduction & Breeding and Epidemic Disease Research, Animal Genetic Engineering Key Laboratory of Haikou, College of Tropical Agriculture and Forestry, Hainan University, Haikou, China

**Keywords:** *FCGBP*, goat bronchial epithelial cells, inflammation, *Pasteurella multocida*, RNA-seq

An incorrect **Funding** statement was provided.

The correct funders are: the Key Research and Development Program of China (2023YFF1000903); China Agriculture Research System of MOF and MARA (CARS-38); Hainan Innovation Center for Academician of Jin Ningyi.

The correct funding statement reads:

The author(s) declared that financial support was received for this work and/or its publication. This work was financially supported by the Key Research and Development Program of China (2023YFF1000903); China Agriculture Research System of MOF and MARA (CARS-38); and Hainan Innovation Center for Academician of Jin Ningyi.

The original version of this article has been updated.

