# Supplementary material for: Dysregulated interferon signaling and hyperinflammation upon FCGBP knockdown in goat bronchial epithelial cells infected with Pasteurella multocida
Source: Front Vet Sci. 2026 Mar 2;13:1761935. doi: 10.3389/fvets.2026.1761935 (PMC12989410; doi:10.3389/fvets.2026.1761935)
Supplement: Supplementary file 1 [file Data_Sheet_1.docx]

Supplementary Material

# Supplementary Figures and Tables

## Supplementary Table

Table S1 Primer sequences for PCR and qRT-PCR

| Gene | GeneID | Primer sequence（5’-3’） |
| --- | --- | --- |
| ISG15 | 102169982 | F: AAGCAGTTCATCGCCCAGAA  R: AGACAGCCAGAATTGGTCCG |
| RSAD2 | 102190797 | F:GCCATCTCCTGTGACAGCTT  R:GCGCGTTGATCTGTTCCTTC |
| TRANK1 | 102182638 | F: TCTTGCCCTGCTTTGATCGT  R: TGAGCCACTGCACTAACCTG |
| IFIH1 | 102183188 | F: GCAGCCAAATCTGGAGGTCT  R: CCCATGGTGCCTGAATCACT |
| DDX58 | 102183788 | F: CTTCAGACTCACTGCCCCAG  R: GACGTGTCGAGAGAAGCACA |
| IMP3 | 102190464 | F: CTGGAGCTCTGCGATTTCGT  R: CGAGTCAACCCAGGTGACAA |
| RRP9 | 102174253 | F: CACAACAATTCTGTGCGGCT  R: AGCACTGGAGAACTTGAGGC |
| FADD | 102170760 | F: GAGGAGAAGTACCCCCGGAA  R: TGAGATCTGCCACCACGTTT |
| PIK3CA | 102168391 | F: GGTTTTGCTGTTCGGTGCTT  R: CCAAAAGCAGGCCAAACCTC |
| COL1A2 | 100860940 | F: GACTTGAGACTCAGCCACCC  R: TGCAGGTTTCGCCAGTAGAG |
| FCGBP | 102185859 | F:TGTAACTATGTGCTGGCATCAACC  R:GTTCTCGTTCTTGGTGGTGACAG |

Table S2 RNA-seq data filtering Statistics table

| Sample | RawDatas | CleanData(%) | Adapter(%) | LowQuality(%) | polyA(%) | N(%) |
| --- | --- | --- | --- | --- | --- | --- |
| C-1 | 48570984 | 48463882 (99.78%) | 19230 (0.04%) | 46124 (0.09%) | 0 (0.00%) | 41748 (0.09%) |
| C-2 | 45843548 | 45729016 (99.75%) | 20022 (0.04%) | 47470 (0.10%) | 0 (0.00%) | 47040 (0.10%) |
| C-3 | 53842760 | 53719378 (99.77%) | 20774 (0.04%) | 37644 (0.07%) | 0 (0.00%) | 64964 (0.12%) |
| CP-1 | 56387632 | 56206846 (99.68%) | 23532 (0.04%) | 57840 (0.10%) | 0 (0.00%) | 99414 (0.18%) |
| CP-2 | 54813648 | 54672402 (99.74%) | 23060 (0.04%) | 48128 (0.09%) | 0 (0.00%) | 70058 (0.13%) |
| CP-3 | 55944854 | 55789122 (99.72%) | 20268 (0.04%) | 55252 (0.10%) | 0 (0.00%) | 80212 (0.14%) |
| NC-1 | 48004040 | 47905080 (99.79%) | 22612 (0.05%) | 41760 (0.09%) | 0 (0.00%) | 34588 (0.07%) |
| NC-2 | 53125130 | 53013674 (99.79%) | 22676 (0.04%) | 48968 (0.09%) | 0 (0.00%) | 39812 (0.07%) |
| NC-3 | 42750406 | 42666434 (99.80%) | 15938 (0.04%) | 38838 (0.09%) | 0 (0.00%) | 29196 (0.07%) |
| NCP-1 | 39208914 | 39128130 (99.79%) | 17518 (0.04%) | 33562 (0.09%) | 0 (0.00%) | 29704 (0.08%) |
| NCP-2 | 39666694 | 39602810 (99.84%) | 16426 (0.04%) | 22662 (0.06%) | 0 (0.00%) | 24796 (0.06%) |
| NCP-3 | 36373356 | 36309964 (99.83%) | 14832 (0.04%) | 22364 (0.06%) | 0 (0.00%) | 26196 (0.07%) |
| F-1 | 44977110 | 44882890 (99.79%) | 21052 (0.05%) | 40886 (0.09%) | 0 (0.00%) | 32282 (0.07%) |
| F-2 | 46986746 | 46893740 (99.80%) | 18466 (0.04%) | 42748 (0.09%) | 0 (0.00%) | 31792 (0.07%) |
| F-3 | 40520838 | 40449932 (99.83%) | 14762 (0.04%) | 33352 (0.08%) | 0 (0.00%) | 22792 (0.06%) |
| FP-1 | 36792406 | 36725418 (99.82%) | 17554 (0.05%) | 23438 (0.06%) | 0 (0.00%) | 25996 (0.07%) |
| FP-2 | 41566160 | 41484134 (99.80%) | 16632 (0.04%) | 32546 (0.08%) | 0 (0.00%) | 32848 (0.08%) |
| FP-3 | 39049224 | 38968494 (99.79%) | 18184 (0.05%) | 34634 (0.09%) | 0 (0.00%) | 27912 (0.07%) |

Table S3 RNA-seq data comparison table with reference genome

| Sample | Total | Unmapped(%) | Unique_Mapped(%) | Multiple_Mapped(%) | Total_Mapped(%) |
| --- | --- | --- | --- | --- | --- |
| C-1 | 48439984 | 1613721 (3.33%) | 44898980 (92.69%) | 1927283 (3.98%) | 46826263 (96.67%) |
| C-2 | 45708290 | 1630471 (3.57%) | 42330074 (92.61%) | 1747745 (3.82%) | 44077819 (96.43%) |
| C-3 | 53690046 | 1675304 (3.12%) | 49880212 (92.90%) | 2134530 (3.98%) | 52014742 (96.88%) |
| CP-1 | 56177604 | 1829345 (3.26%) | 52041428 (92.64%) | 2306831 (4.11%) | 54348259 (96.74%) |
| CP-2 | 54643184 | 1906515 (3.49%) | 50518773 (92.45%) | 2217896 (4.06%) | 52736669 (96.51%) |
| CP-3 | 55756948 | 1750135 (3.14%) | 51753488 (92.82%) | 2253325 (4.04%) | 54006813 (96.86%) |
| NC-1 | 47857574 | 1802839 (3.77%) | 44035578 (92.01%) | 2019157 (4.22%) | 46054735 (96.23%) |
| NC-2 | 52954064 | 1989832 (3.76%) | 48636178 (91.85%) | 2328054 (4.40%) | 50964232 (96.24%) |
| NC-3 | 42615752 | 1590093 (3.73%) | 39146964 (91.86%) | 1878695 (4.41%) | 41025659 (96.27%) |
| NCP-1 | 39075466 | 1512041 (3.87%) | 36054523 (92.27%) | 1508902 (3.86%) | 37563425 (96.13%) |
| NCP-2 | 39536586 | 1441869 (3.65%) | 36535402 (92.41%) | 1559315 (3.94%) | 38094717 (96.35%) |
| NCP-3 | 36254134 | 1302402 (3.59%) | 33545166 (92.53%) | 1406566 (3.88%) | 34951732 (96.41%) |
| F-1 | 44836320 | 2781774 (6.20%) | 40128820 (89.50%) | 1925726 (4.30%) | 42054546 (93.80%) |
| F-2 | 46832416 | 2682137 (5.73%) | 41986961 (89.65%) | 2163318 (4.62%) | 44150279 (94.27%) |
| F-3 | 40398428 | 2247065 (5.56%) | 36357432 (90.00%) | 1793931 (4.44%) | 38151363 (94.44%) |
| FP-1 | 36674670 | 2209267 (6.02%) | 33034908 (90.08%) | 1430495 (3.90%) | 34465403 (93.98%) |
| FP-2 | 41425742 | 2460331 (5.94%) | 37334944 (90.12%) | 1630467 (3.94%) | 38965411 (94.06%) |
| FP-3 | 38911390 | 2541106 (6.53%) | 34731065 (89.26%) | 1639219 (4.21%) | 36370284 (93.47%) |

## Supplementary Figure
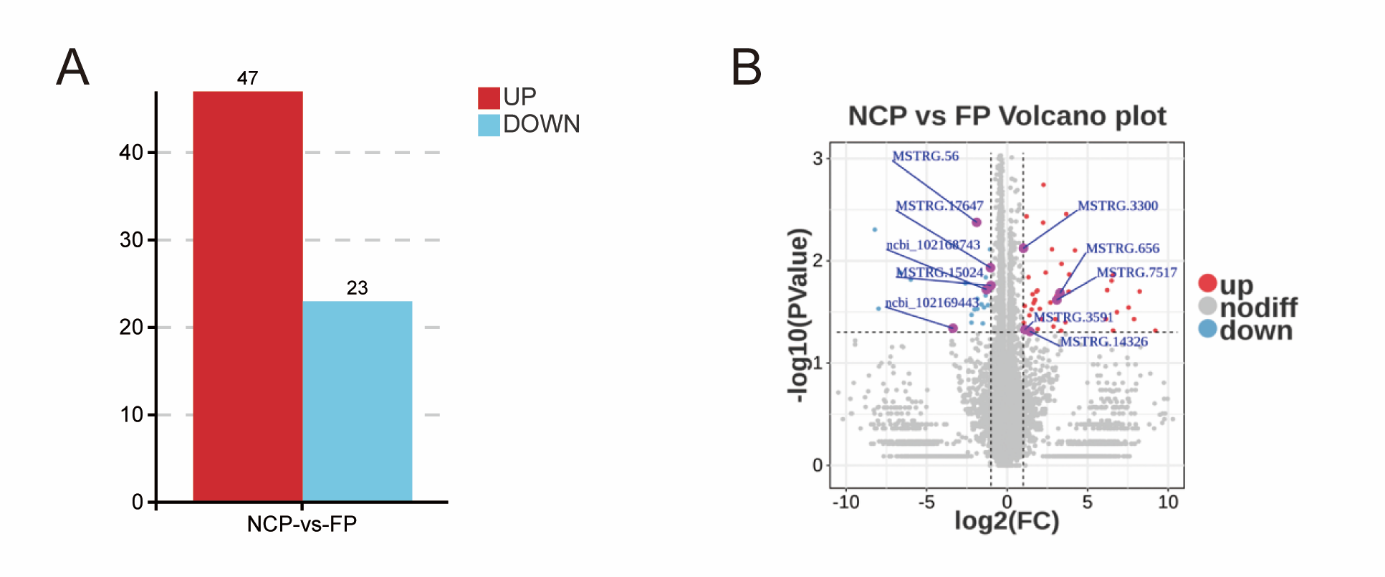


Fig. S1 (A) Bar chart of differentially expressed genes (DEGs) between the NCP and FP groups. (B) Volcano plots of DEGs in NCP vs FP.


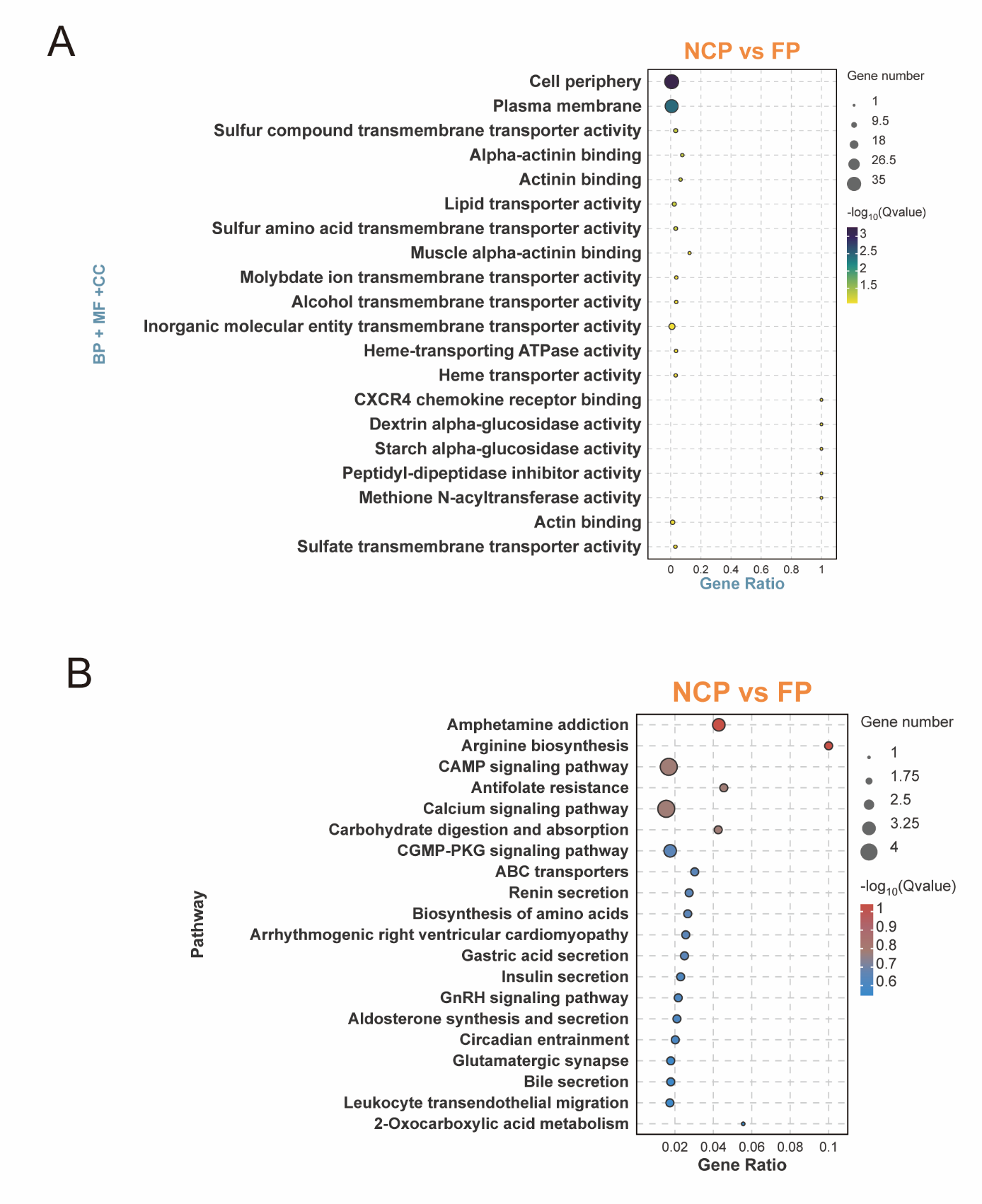


Fig. S2 (A) GO pathways enriched in DEGs from the NCP vs FP group (B) KEGG pathway enrichment analysis of differentially expressed genes (DEGs) in the comparison of NCP vs FP.


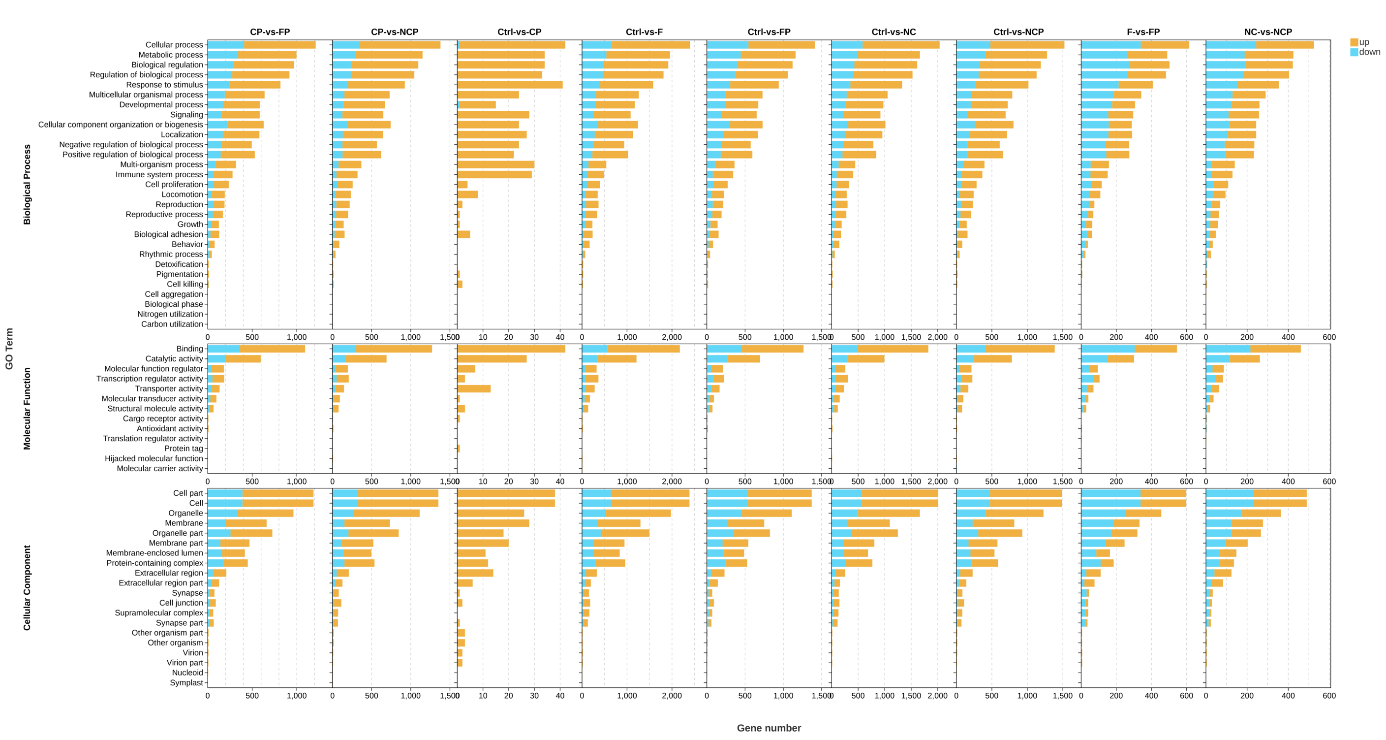


Fig. S3 GO Secondary Category Bar Chart
